# Relationship between average daily rehabilitation time and decline in instrumental activity of daily living among older patients with heart failure: A preliminary analysis of a multicenter cohort study, SURUGA-CARE

**DOI:** 10.1371/journal.pone.0254128

**Published:** 2021-07-02

**Authors:** Michitaka Kato, Yuji Mori, Daisuke Watanabe, Hiroshige Onoda, Keita Fujiyama, Masahiro Toda, Kazuya Kito

**Affiliations:** 1 Faculty of Health Science, Department of Shizuoka Physical Therapy, Tokoha University, Shizuoka, Japan; 2 Department of Rehabilitation, Shizuoka Medical Center, Shizuoka, Japan; 3 Department of Rehabilitation, Juntendo University Shizuoka Hospital, Shizuoka, Japan; 4 Department of Rehabilitation, Shizuoka City Hospital, Shizuoka, Japan; 5 Department of Rehabilitation, Fujinomiya City General Hospital, Shizuoka, Japan; 6 Department of Rehabilitation, Hamamatsu University Hospital, Shizuoka, Japan; Maastricht University Medical Center, NETHERLANDS

## Abstract

**Background:**

Limitation of instrumental activity of daily living (IADL) is independently associated with an adverse prognosis in older heart failure (HF) patients.

**Aims:**

This multicenter study aims to examine the relationship between average daily rehabilitation time (ADRT) and risk of IADL decline during acute hospitalization in older patients with HF.

**Methods:**

Four hundred eleven older patients who were hospitalized due to acute HF and underwent rehabilitation were divided into three groups based on the tertile of the ADRT: short, intermediate, and long groups. IADL was assessed by the National Center for Geriatrics and Gerontology Activities of Daily Living (NCGG-ADL) scale. Change in NCGG-ADL (Δ NCGG-ADL) was calculated by subtracting the pre-hospitalization score from the at-discharge score and IADL decline was defined as Δ NCGG-ADL < = −1 point. Logistic regression analysis was carried out examining the association between ADRT and occurrence of IADL decline.

**Results:**

The ADRT was 23.9, 32.0, and 38.6 minutes in short, intermediate, and long group, respectively. The proportion of patients with IADL decline during hospitalization was 21% among all subjects and short group had the highest proportion of IADL decline (33%) and long group had the lowest proportion (14%). The long group had significantly lower odds of IADL decline compared with the short group (OR:0.475, 95% CI:0.231–0.975, P = 0.042). Among the items of NCGG-ADL scale, significant decreases in the “go out by oneself”, “travel using a bus or train”, “shop for necessities”, “vacuum”, and “manage medication” were observed at discharge compared to pre-hospitalization in the short group (p<0.01, p<0.01, p<0.01, p<0.05, and p<0.05).

**Conclusions:**

The present study demonstrated that short of ADRT may be associated with the risk of IADL decline during hospitalization in older patients with HF.

## Introduction

With the rapid aging of the population in Japan, the numbers of older patients with heart failure (HF) are increasing dramatically [1]. Older HF patients have high mortality and hospital readmission rates resulting in increased medical expenses [2]. Therefore, improving the long-term prognosis and reducing the number of hospital readmissions are urgent issues that need to be addressed as goals in the field of HF treatment.

Increasing functional capacity is important to improve healthy life expectancy and reduce hospital readmissions [3]. Maintaining the ability to perform activities of daily living (ADLs) is also associated with favorable prognosis in older HF patients [4,5]. Decline in ADL can progress over time, and a large community cohort study has reported that majority of older HF patients have difficulty with one or more basic ADLs [5]. Instrumental activity of daily living (IADL) is commonly confused with basic ADL, but while ADL describes fundamental skills to independently care for oneself, IADL requires more complex skills related to independent living. IADLs include cooking, cleaning, transportation, laundry, and managing finances [6]. In 2015, about 13% of male and 21% of female aged 65 years or older were living alone in Japan [7]. Given that the number of older people living alone is increasing, not just the ADL, but the IADL independence is necessary for them to continue living independently in the community. A recent study has found that older age, female gender, lower education, physical inactivity, frailty, having two or more chronic diseases, presence of depression, polypharmacy, poor self-perception of health and lower network satisfaction are associated with IADL limitation [8]. Another study reported that IADL limitation was independently associated with an adverse prognosis in older HF patients [9]. Therefore, maintaining not just the ADL but also the IADL may be crucial in improving the prognosis of older patients with HF. The Japanese Circulation Society has established a standardized cardiac inpatient rehabilitation program to promote appropriate rehabilitation implementation and prevent decline in functional capacity, ADL and/or IADL in acute clinical settings [10]. However, the relationship between average daily rehabilitation time (ADRT) and the ADL or IADL decline is unknown.

This multicenter study aims to examine the relationship between ADRT and IADL decline during acute hospitalization, in older patients with HF.

## Methods

### Study design

The present study was a preliminary analysis of a multicenter cohort study, SURUGA-CARE. SURUGA-CARE study is an ongoing multicenter, prospective cohort study and a collaboration work of 5 hospitals and 1 university in Shizuoka, Japan. SURUGA-CARE study started in March 2017 and is expected to be completed in March 2021. This study was prepared in accordance with the standards set forth by the Strengthening the Reporting of Observational Studies in Epidemiology (STROBE) statement [11].

### Patients

The inclusion criteria for this study was HF patients who underwent rehabilitation between March 2017 and October 2019 at one of the five hospitals participating in this study. We included 1392 patients who were hospitalized due to acute HF. The exclusion criteria included the presence of one or more of the following: age < 65 years old, death during hospitalization, bedridden or extremely low ADL before hospitalization, severe dementia (a score on the Mini-Mental State Examination (MMSE) < 17 points), and IADL score (described later) = 0 point. We also excluded patients who had hospital stay < = 5 days and/or the number of days for rehabilitation < = 3 days because the effect of rehabilitation among these patients is questionable. In addition, patients who had hospital stay > = 3 months were also excluded because they may have social or other health problems that prevented them from getting discharged.

### Ethics

The study design was approved by the ethics committee of Shizuoka Medical Center (28–10) and the institutional review boards of all participating hospitals. All patients were informed about the study and provided consent.

### Acute-phase and early recovery phase rehabilitation during hospitalization

All the participating hospitals provided standardized cardiac inpatient rehabilitation according to the guidelines by the Japanese Circulation Society and the Japanese Association of Cardiac Rehabilitation [10,12]. The standardized rehabilitation began with an individual low-intensity exercise training on the bed for acute HF patients who were hemodynamically stable and asymptomatic at rest even when they were receiving intravenous infusion. Then, the stage of the program was gradually advanced to include standing at the bedside and walking around the bedside and up to 80 meters within 5–10 minutes on the ward. After the ambulation program, patients were prescribed an individual or group exercise program consisting of walking and endurance training with a Borg scale of 11 to 13 or 40–60% of the heart rate reserve and a resistance training with a Borg scale < 13 (upper limb exercise at 30–40% of 1 repetition maximum (RM) and lower limb exercise of 50–60% of 1RM) [8]. Walking and endurance training was done using a stationary bike or treadmill and was gradually increased from approximately 5 to 20 minutes. The resistance training was for upper and lower limbs using rubber band, muscle training machine, or own weight. Progress in rehabilitation was carried out according to criteria for contraindications and precautions for exercise training in patients with HF [10]. The target daily rehabilitation time was 20 to 60 minutes, depending on the patient’s hemodynamic status, HF symptoms, rehabilitation needs, and endurance. Rehabilitation was provided 4 or 5 times per week at each hospital, and continued until patients were discharged. The target rehabilitation time per day and frequency per week were determined by each patient’s physician and physical therapist. The content of exercise therapy was coordinated among facilities as much as possible in advance.

### Objective variable

The objective variable in this study was IADL decline during hospitalization. The IADL was assessed by the National Center for Geriatrics and Gerontology Activities of Daily Living (NCGG-ADL) scale, which is a simple and highly reliable tool [11,12]. The NCGG-ADL contains questions about 13 daily activities: (1) cut toenails, (2) go out by oneself, (3) travel using a bus or train, (4) shop for necessities, (5) pay bills, (6) look up a telephone number, (7) vacuum, (8) manage finances, (9) manage medications, (10) manage a house key, (11) prepare meals, (12) use a microwave, and (13) use a gas stove [13]. Patients reported their ability to conduct each of the 13 activities independently, using a simple dichotomous rating (yes/no). Additionally, physical therapists carefully assessed patients’ physical and cognitive functions and ADL levels, which was also taken into account when determining which activities patients could perform. The score was calculated by summing the number of “yes” responses (0–13) with higher scores indicating higher ability in IADL. In our study, IADL was measured twice using the NCGG-ADL scale. First, to assess pre-hospitalization status, we evaluated all subjects as soon as possible after admission, asking them to recall whether they were able to perform the activities listed in the NCGG-ADL prior to admission (pre-hospitalization score). Then, we evaluated all subjects again the day before discharge (at-discharge score). The change in NCGG-ADL (Δ NCGG-ADL) was calculated by subtracting the pre-hospitalization score from the at-discharge score. The IADL decline was defined as Δ NCGG-ADL < = −1 point in this study [13].

### Other variables

Other clinical variables including age, sex, body mass index (BMI), comorbidities, devices, severity of HF (New York Heart Association functional classification [NYHA]) before hospitalization, biochemical data, and echocardiographic data were obtained from medical records at the time of hospitalization. MMSE was assessed as a parameter of cognitive function after introduction of rehabilitation. The Barthel Index (BI) was assessed as a parameter of ADL at discharge. As a physical function assessment, we measured the Short Physical Performance Battery (SPPB), usual gait speed, and leg strength at discharge [14,15]. The total rehabilitation time was calculated by summing all rehabilitation time including low-intensity exercise training on the bed and acute phase ambulation program during hospitalization. The ADRT was calculated by dividing the total rehabilitation time by the total number of days of rehabilitation during hospitalization.

### Statistical analysis

Data are presented as median [interquartile range] or mean ± standard deviation for continuous variables and percentages for categorical variables.

The included patients were divided into three groups based on the tertile of the ADRT: short, intermediate, and long groups. Baseline characteristics and other parameters were compared among the three groups using the Kruskal-Wallis or Fisher’s exact test, whichever that was more appropriate, and a Steel-Dwass post-hoc analysis was also conducted if indicated. Then, patients were divided into two categories based on the presence of IADL decline: IADL decline or IADL maintained. Baseline characteristics were compared between the two groups using Mann-Whitney U test or Fisher’s exact test, whichever that was more appropriate. Univariate and multivariate logistic regression analyses were conducted to assess the odds of IADL decline by the ADRT, using the short group as the reference group. For multivariate analyses, the variables identified to be significantly associated with IADL decline in univariate analysis were used as covariates. Odds ratios (OR), 95% confidence intervals (CI), and significance levels were calculated. The Wilcoxon signed rank test was used to assess statistical differences in each item of NCGG-ADL measured pre-hospitalization and at discharge.

All analyses were carried out using SPSS 25.0 for Windows (SPSS Statistics, IBM, Tokyo, Japan). In all analyses, 2-tailed P values < 0.05 were considered statistically significant.

## Results

**Fig 1:**
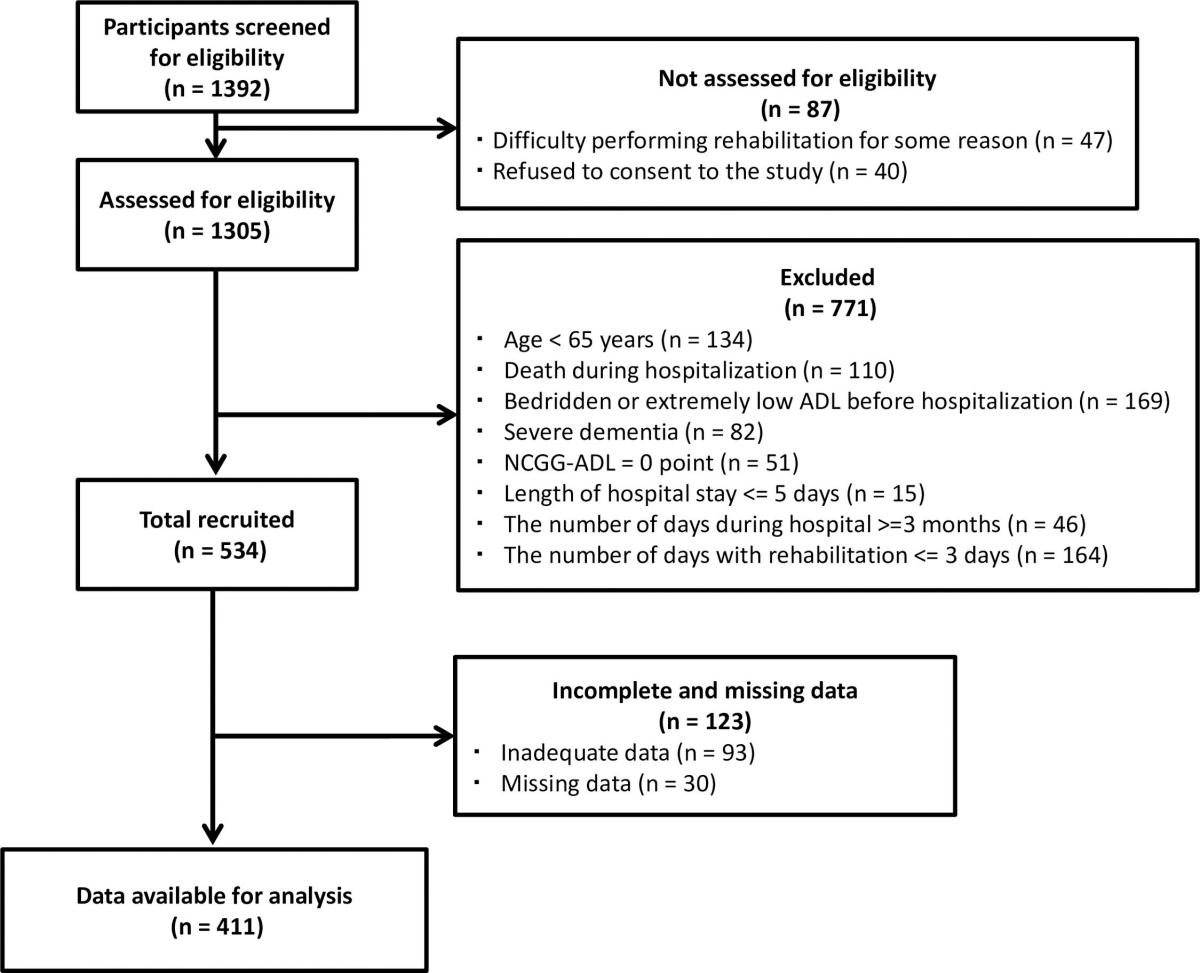
Flow diagram. ADL, activity of daily living; NCGG-ADL, the National Center for Geriatrics and Gerontology Activities of Daily Living.

Participant flow of the study is shown in Fig 1. Of the 1392 consecutive HF patients who underwent rehabilitation, a total of 1305 were deemed eligible in this study; however, 771 were excluded because they met one or more of the exclusion criteria and 123 patients were later excluded due to incomplete or missing data. Finally, 411 patients were included in the analyses.

Table 1 shows a comparison of patient characteristics of the older hospitalized HF patients among the short, intermediate, and long groups (n = 139, 139, and 133, respectively). When comparing the short group’s values with the intermediate group’s, age was significantly higher (P<0.05), and albumin (Alb), MMSE, and ADRT were significantly lower (P<0.05, P<0.05, P<0.001, respectively). When comparing the intermediate group with the long group, HDL-C was significantly higher (P<0.05), and ADRT was significantly shorter (P<0.001). When comparing the long group’s values with the short group’s, MMSE and ADRT were significantly higher (P<0.01 and P<0.001). ADRT was 23.9 [20.0–25.7], 32.0 [30.0–34.2], and 38.6 [36.6–40.1] minutes in short, intermediate, and long group, respectively.

**Table 1:** Comparison of patient characteristics among the 3 groups based on average daily rehabilitation time.

	short group	intermediate group	long group	*P-value*			
				Kruskal-Wallis/Fisher’s exact test	Post-Hoc		
	(n = 139)	(n = 139)	(n = 133)		S vs I	I vs L	L vs S
Age (years)	84 [78–89]	82 [77–86]	82 [77–86]	< 0.05	< 0.05	―	―
Male, n (%)	68 (48)	67 (48)	70 (53)	0.620	―	―	―
BMI (kg/m^2^)	19.8 [17.9–22.2]	20.4 [18.5–22.5]	20.4 [18.1–23.1]	0.302	―	―	―
HT, n (%)	103 (74)	97 (70)	84 (63)	0.172	―	―	―
DM, n (%)	61 (43)	67 (48)	48 (36)	0.130	―	―	―
DL, n (%)	49 (35)	51 (37)	41 (31)	0.521	―	―	―
CKD, n (%)	58 (41)	47 (34)	41 (31)	0.222	―	―	―
COPD, n (%)	15 (10)	8 (6)	13 (10)	0.398	―	―	―
CVA, n (%)	26 (18)	22 (16)	19 (14)	0.679	―	―	―
AF, n (%)	56 (40)	54 (39)	51 (38)	0.963	―	―	―
Fragility fracture	19 (14)	14 (10)	15 (11)	0.386	―	―	―
ICD/CRT-D, n (%)	3 (2)	6 (4)	10 (8)	0.104	―	―	―
NYHA, n (%) (I/II/III/IV)	42(30)/45(32)/29(21)/23(17)	41(29)/52(37)/30(22)/16(12)	36(27)/44(33)/33(25)/20(15)	0.793	―	―	―
Alb, mg/dL	3.4 [3.1–3.7]	3.6 [3.2–3.9]	3.5 [3.2–3.7]	< 0.05	< 0.05	―	―
Cr, mg/dL	1.26 [0.89–1.85]	1.18 [0.92–1.83]	1.15 [0.82–1.63]	0.304	―	―	―
LDL-C, mg/dL	88 [69–107]	93 [71–106]	84 [66–110]	0.318	―	―	―
HDL-C, mg/dL	47 [40–58]	51 [42–64]	46 [37–55]	<0.05	―	< 0.05	―
HbA1c, %	6.0 [5.7–6.8]	6.0 [5.7–6.6]	6.0 [5.6–6.6]	0.713	―	―	**―**
Hb, g/dL	10.8 [9.8–12.2]	11.3 [9.7–12.8]	11.1 [9.9–12.5]	0.913	―	―	―
BNP, pg/dL	653 [413–1117]	783 [419–1372]	675 [451–1109]	0.192	―	―	―
LVEF, %	46.6 [33.0–62.1]	45.4 [31.6–57.1]	45.0 [33.0–58.9]	0.329	―	―	―
LAD, mm	43.0 [38.0–47.8]	43.3 [40.1–47.8]	44.0 [39.1–48.2]	0.502	―	―	―
MMSE, points	24 20–26]	25 [22–27]	25 [22–28]	< 0.01	< 0.05	―	< 0.01
History of hospitalization for HF, n (%)	68 (49)	61 (44)	52 (39)	0.251	―	―	―
ADRT, minutes/day	23.9 [20.0–25.7]	32.0 [30.0–34.2]	38.6 [36.6–40.1]	< 0.001	< 0.001	< 0.001	< 0.001

ADRT, average daily rehabilitation time; BMI, body mass index; HT, hypertension; DM, diabetes mellitus; DL, dyslipidemia; CKD, chronic kidney disease; COPD, chronic obstructive pulmonary disease; CVA, cerebrovascular accident; AF, ICD, implantable cardioverter-defibrillator; CRT-D, cardiac resynchronization therapy defibrillator; NYHA, New York Heart Association functional classification; atrial fibrillation; Alb, Albumin; Cr, creatinine; LDL-C, low density lipoprotein cholesterol; HDL-C, high density lipoprotein cholesterol; HbA1c, glycohemoglobin; Hb, hemoglobin; BNP, brain natriuretic peptide; LVEF, left ventricular ejection fraction; LAD, left atrial dimension; MMSE, Mini Mental State Examination; HF, heart failure; S, short; I, intermediate, L, long.

Values are presented as median [interquartile range] or as n (%).

Number of days from hospitalization to the start of rehabilitation, number of days of rehabilitation, length of stay, Δ NCGG-ADL, and ADL and physical function assessments at discharge for each group are shown in Table 2. Number of days of rehabilitation during hospitalization and length of hospital stay were not significantly different among groups. Both the pre-hospitalization and at-discharge NCGG-ADL scores were significantly lower in the short group compared with the intermediate and long groups (P<0.01 for all). Δ NCGG-ADL in short group was significantly larger compared with intermediate and long groups (P<0.01 and P<0.001).

**Table 2:** Number of days of rehabilitation, length of hospital stay, and Δ NCGG-ADL among the 3 groups.

	short group	intermediate group	long group	*P-value*			
				Kruskal-Wallis	Post-Hoc		
	(n = 139)	(n = 139)	(n = 133)		S vs I	I vs L	L vs S
Number of days from hospitalization to the start of rehabilitation	6 [4–9]	5 [3–7]	5 [4–8]	0.021	0.01	―	―
Number of days of rehabilitation during hospitalization, days	8 [6–11]	9 [5–12]	8 [6–11]	0.876	―	―	―
Length of hospital stay, days	22 [17–30]	21 [16–29]	20 [16–26]	0.135	―	―	―
Pre-hospitalization NCGG-ADL, points	10.5 [6–13]	12 [8–13]	12 [9–13]	< 0.001	< 0.01	―	< 0.01
At-discharge NCGG-ADL, points	8.5 [4–12]	12 [8–13]	12 [8–13]	< 0.001	< 0.01	―	< 0.01
Δ NCGG-ADL, points	0 [–1–0] -0.94 ± 2.08	0 [0–0] -0.46 ± 2.10	0 [0–0] -0.26 ± 1.58	< 0.001	< 0.01	―	< 0.001
At-discharge BI, points	85 [70–95]	95 [80–100]	100 [80–100]	< 0.001	< 0.01	―	0.02
At-discharge SPPB, points	7 [4–9.8]	9 [6–11]	9 [6–11]	0.006	0.02	―	0.02
At-discharge usual gait speed, m/s	0.59 [0.45–0.77]	0.77 [0.59–0.92]	0.71 [0.55–0.85]	< 0.001	< 0.01	―	0.04
At-discharge leg strength (%BW)	0.31 [0.24–0.38]	0.30 [0.24–0.39]	0.32 [0.23–0.41]	―	―	―	―

NCGG-ADL, National Center for Geriatrics and Gerontology-Activities of Daily Living Scale; ΔNCGG-ADL, changes in NCGG-ADL during hospitalization; BI, Barthel index; SPPB, the Short Physical Performance Battery; BW, body weight; S, short; I, intermediate; L, long. Values are presented as median [interquartile range] or mean ± standard deviation.

**Fig 2:**
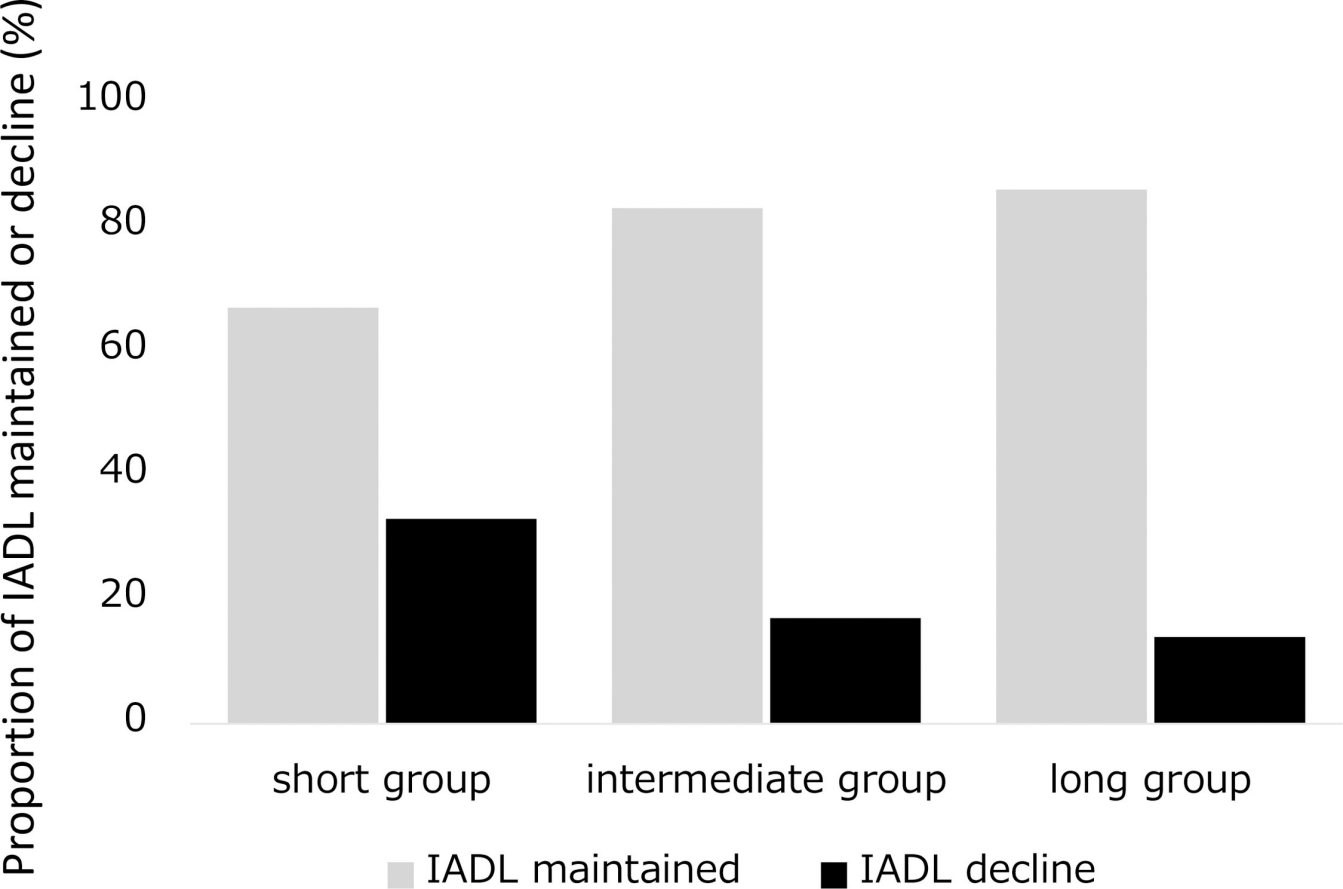
Proportion of IADL maintained and decline among the 3 groups. IADL, instrumental activity of daily living.

Fig 2 shows the proportion of patients categorized as IADL maintained and IADL decline among the 3 groups. The proportion of patients with IADL decline was 21% among all subjects in this study. Short group had the highest proportion of IADL decline (33%) and long group had the lowest proportion (14%).

Table 3 shows a comparison of patient characteristics between the IADL maintained and decline categories. In the IADL decline category, age and left ventricular ejection fraction (LVEF) were significantly higher (P<0.001, P = 0.025, respectively), and Alb and MMSE were significantly lower (P = 0.002, P<0.001, respectively) compared with the IADL maintained category. In addition, patients with IADL decline during hospitalization had a significantly lower pre-hospitalization IADL compared with those in the IADL maintained category (P<0.001).

**Table 3:** Comparison of patient characteristics between IADL maintained and decline categories.

	IADL maintained (n = 320)	IADL decline (n = 91)	*P-value*
Age, years	82 [76–86]	85 [80–89]	< 0.001
Male, n (%)	164 (51)	42 (46)	0.475
BMI, kg/m^2^	20.1 [18.2–22.6]	20.1 [17.6–23.0]	0.600
HT, n (%)	223 (69)	63 (68)	0.897
DM, n (%)	131 (40)	46 (50)	0.148
DL, n (%)	110 (34)	32 (35)	0.900
CKD, n (%)	115 (35)	33 (36)	0.902
COPD, n (%)	32 (10)	5 (5)	0.296
CVA, n (%)	51 (15)	17 (18)	0.522
AF, n (%)	122 (38)	40 (44)	0.275
Fragility fracture	38 (12)	10 (11)	0.856
ICR/CRT-D, n (%)	8 (5)	1 (1)	0.097
NYHA, n (%) (I/II/III/IV)	86(27)/108 (34)/75(23)/51(16)	34(37)/30(33)/18(20)/9(10)	0.206
Alb, mg/dL	3.5 [3.2–3.8]	3.2 [2.8–3.5]	0.002
Cr, mg/dL	1.17[0.87–1.81]	1.28 [0.90–2.06]	0.382
LDL-C, mg/dL	89 [70–110]	86 [69.5–103.5]	0.663
HDL-C, mg/dL	48 [39–60]	46 [36–53]	0.068
HbA1c, %	6.0 [5.7–6.7]	6.1 [5.7–6.7]	0.376
Hb, g/dL	11.2 [9.8–12.8]	10.8 [9.4–12.3]	0.084
BNP, pg/mL	734 [415–1313]	627 [483–1097]	0.528
LVEF, %	45 [32–58]	50.5 [35.4–62.3]	0.025
LAD, mm	43.9 [39.6–48.0]	42.2 [38.6–47.3]	0.345
MMSE, points	25 [22–28]	23 [19–25]	< 0.001
History of hospitalization for HF, n (%)	135 (42)	49 (53)	0.0715
Pre-hospitalization NCGG-ADL, points	12 [8–13]	10 [7–12]	< 0.001

IADL, instrumental activity of daily living; BMI, body mass index; HT, hypertension; DM, diabetes mellitus; DL, dyslipidemia; CKD, chronic kidney disease; COPD, chronic obstructive pulmonary disease; CVA, cerebrovascular accident; AF, atrial fibrillation; ICD, implantable cardioverter-defibrillator; CRT-D, cardiac resynchronization therapy defibrillator; NYHA, New York Heart Association functional classification; Alb, Albumin; Cr, creatinine; LDL-C, low density lipoprotein cholesterol; HDL-C, high density lipoprotein cholesterol; HbA1c, glycohemoglobin; Hb, hemoglobin; BNP, brain natriuretic peptide; LVEF, left ventricular ejection fraction; LAD, left atrial dimension; MMSE, Mini Mental State Examination; HF, heart failure.

Values are presented as median [interquartile range] or as n (%).

**Fig 3:**
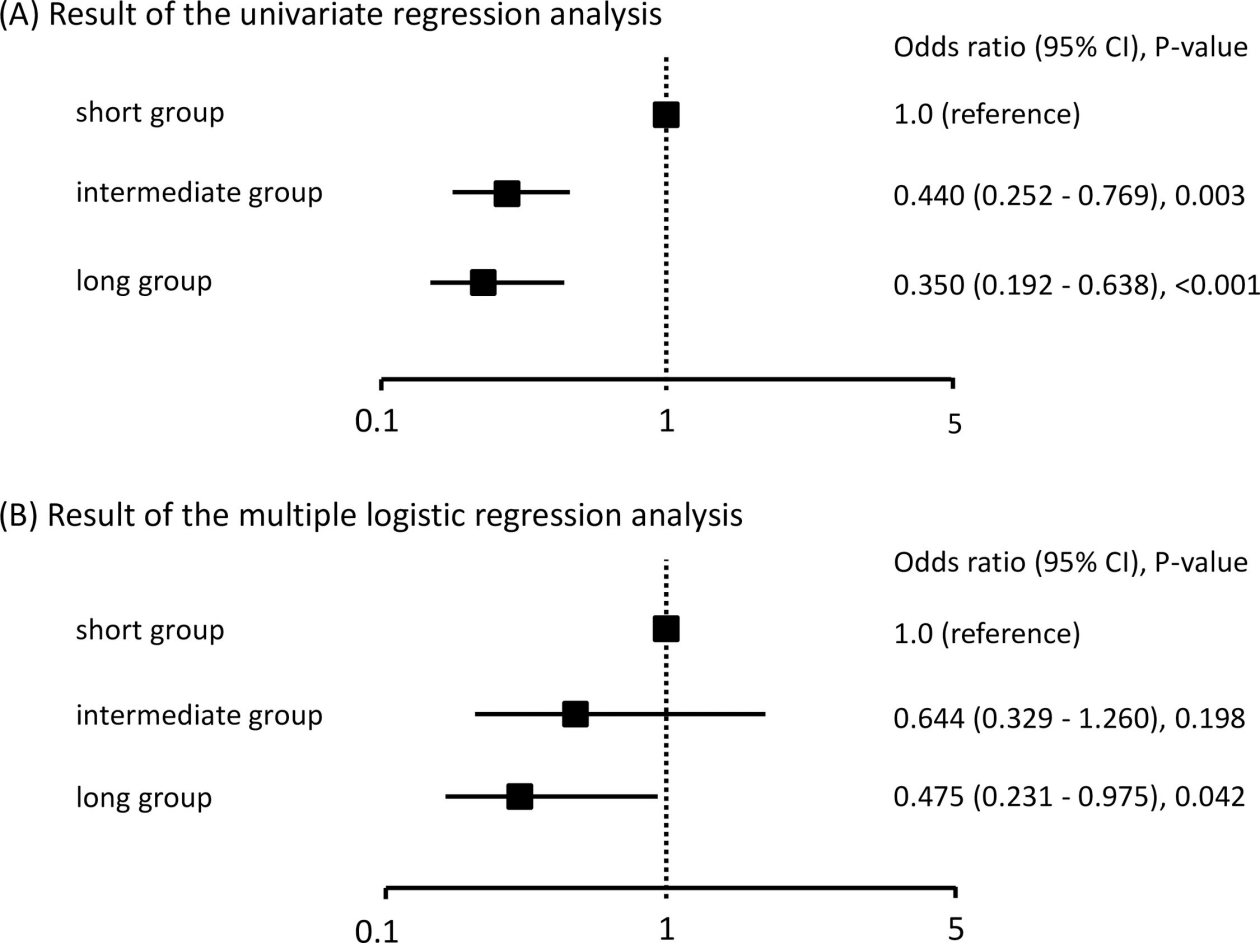
Univariate and multivariate logistic regression analyses of the odds of IADL decline. Multivariate logistic regression analysis was adjusted for age, albumin, left ventricular ejection fraction, Mini-Mental State Examination score, and the pre-hospitalization score on the National Center for Geriatrics and Gerontology Activities of Daily Living scale. CI, confidence interval.

Fig 3 shows the results of the univariate and multiple logistic regression analyses of the odds of IADL decline by ADRT. In the multiple logistic regression analysis model with age, Alb, LVEF, MMSE, and pre-hospitalization NCGG-ADL as covariates, the long group had significantly lower odds of IADL decline compared with the short group (OR and 95% CI: 0.475, 0.231–0.975, P = 0.042).

Table 4 shows the changes in each item of NCGG-ADL. In the short group, significant decreases in the “go out by oneself”, “travel using a bus or train”, “shop for necessities”, “vacuum”, and “manage medication” were observed at discharge compared to pre-hospitalization (p<0.01, p<0.01, p<0.01, p<0.05, and p<0.05). No significant declines were observed in the long group.

**Table 4:** Changes in each item of NCGG-ADL.

		short group		long group	
No.	NCGG-ADL items	pre-hospitalization	at discharge	pre-hospitalization	at discharge
1	cut nails	0.82±0.39	0.82±0.39	0.87±0.34	0.87±0.34
2	go out by oneself	0.71±0.46	0.60±0.49 **	0.85±0.36	0.80±0.40
3	travel using a bus or train	0.60±0.49	0.51±0.50 **	0.79±0.41	0.79±0.41
4	shop for necessities	0.64±0.48	0.53±0.50 **	0.88±0.33	0.84±0.37
5	pay bills	0.68±0.47	0.65±0.48	0.84±0.37	0.82±0.38
6	look up a telephone number	0.90±0.30	0.89±0.32	0.95±0.22	0.95±0.22
7	vacuum	0.68±0.47	0.63±0.49 *	0.89±0.31	0.84±0.37
8	manage finances	0.79±0.41	0.78±0.42	0.93±0.26	0.87±0.34
9	manage medications	0.71±0.46	0.65±0.48 *	0.87±0.34	0.87±0.34
10	manage a house key	0.85±0.36	0.85±0.36	0.90±0.30	0.90±0.30
11	prepare meals	0.72±0.45	0.69±0.46	0.87±0.34	0.85±0.36
12	use a microwave	0.89±0.32	0.86±0.35	0.91±0.28	0.91±0.28
13	use a gas stove	0.74±0.44	0.69±0.46	0.84±0.37	0.80±0.40

* P<0.05, vs. pre-hospitalization within group.

** P<0.01, vs. pre-hospitalization within group.

NCGG-ADL, National Center for Geriatrics and Gerontology-Activities of Daily Living Scale.

## Discussions

The present study showed that 21% of older HF patients had IADL decline during hospitalization and increased ADRT was a significant independent factor associated with lower occurrence of IADL decline. This is the first study to report the relationship between rehabilitation time and the risk of IADL decline in older patients with HF.

In the present study, we evaluated IADL decline based on the NCGG-ADL score, although there are several other available assessment scores that may be more widely used. In 1972, Lowton et al developed the first assessment tool for IADL [16]. Later in 1992, the Tokyo Metropolitan Institute of Gerontology Index of Competence (TMIG-IC), which is more specific to the cultural background and lifestyle aspects of Japan, was reported [17], and has been widely used in Japan. However, since lifestyle has changed significantly in the past decades, it is necessary to use an IADL assessment tool that matches current lifestyle. NCGG-ADL is a new assessment tool for IADL developed in 2013 to match the recent lifestyle changes in Japan. A previous study has demonstrated that a difference of one or more points in the NCGG-ADL among older people was significantly associated with the risk of functional disability in the future [13]. Additionally, we considered that NCGG-ADL is more suitable than other tools for our study population because it assesses not only the ability to go out and do housework, but also the ability to manage medications, which is a crucial factor for HF patients.

Hospital-acquired disability (HAD), defined as loss of independence in ADL and/or IADL following acute hospitalization, has recently been recognized as an important outcome to assess in the older population [18]. HAD affects mid-to-long term prognosis after hospitalization [19]. Recent meta-analysis reported that the prevalence of HAD among older adults is approximately 30% [18]. Jonckers et al investigated the prevalence of HAD in older adults with valvular heart disease and found that 51.9% of their subjects developed HAD [20]. Other studies reported the prevalence of HAD as 41% and 33% among older adults admitted to general medicine and cardiac care unit, respectively [21,22]. In our study, the prevalence of HAD was 21%, which seems lower than these previously reported studies. However, it is difficult to compare the prevalence of HAD among studies because we only assessed the decline in IADL, while some studies assessed the decline only in ADL or both ADL and IADL. There are also other differences between our study and the previous studies, such as disease types, occurrence of surgery, baseline ADL/IADL, method of ADL/IADL assessment, and occurrence of physiotherapist and/or occupational therapist consultation [20–22]. Moreover, there was a large difference between the length of hospital stay. While the length of stay was 23 days in average for our study, it was 4 to 13 days in the previous studies [20–22]. Thus, patients in our study may have undergone more rehabilitation compared to patients in the previous studies, which may have contributed to the lower prevalence of HAD.

When dividing patients into groups based on their ADRT during hospitalization, the long group had significantly lower risk of IADL decline compared to the short group, even after adjusting for age, nutritional status, cardiac function, cognitive function, and disability status. During acute hospitalization, HAD could develop within days for any adult, but is more common among older patients and people with depression, cognitive impairment, enforced bed rest, and/or poor nutrition [19,23,24]. Similarly, our study also showed that patients with good cognitive function (high MMSE score) had lower odds of IADL decline (OR and 95% CI: 0.89, 0.82–0.98, P = 0.016) and those with good nutrition status (high Alb) tended to have lower odds of IADL decline (OR and 95% CI: 0.50, 0.24–1.04, P = 0.06) in the multiple logistic regression analyses. HAD is believed to be a result of physical inactivity during hospitalization [21], and therefore, early ambulation or stepwise exercise program to increase physical activity may play an important role in preventing ADL and/or IADL decline [25]. In a previous observational study, walking more than 900 steps per day during hospitalization was associated with prevention of HAD after adjusting for functional status and other major personal risk factors [26]. A randomized controlled trial demonstrated that a simple inpatient exercise program consisting of walking and rising from a chair decreased the risk of developing HAD in acutely hospitalized, very old patients [27]. These results imply that there is a relationship between physical intervention or amount of physical activity during hospitalization and the risk of HAD. In addition, ADRT seems to correlate positively with improved physical function and ADL after stroke [28]. Yosef-Brauner et al examined the effect of the number of physical therapy (PT) sessions on muscle strength and physical function and showed that PT twice a day was better than once a day to facilitate the recovery process in patients who suffer from intensive care unit-acquired weakness [29]. These findings, although with different patient types and/or settings, are consistent with our results, suggesting that increased physical activity during hospitalization contributes to the prevention of functional impairment and disability during hospitalization.

The standardized cardiac inpatient rehabilitation program by the Japanese Circulation Society encourages HF patients to continue and increase walking training and/or endurance training using a stationary bike or treadmill after ambulation program around the bedside and on the ward [10]. Although the ideal duration of the rehabilitation program to prevent impairment and disability during hospitalization is unknown, older HF patients with stable hemodynamic status might benefit from longer programs, such as those with an average daily duration of 40 minutes or more. In our study, only the long group, and not the intermediate group, had a significantly lower risk of IADL decline compared to the short group, and the median values for the ADRT were 32.0 and 38.6 minutes per day for the intermediate and long groups, respectively. The underlying cause for shorter ADRT for some patients in our study is unclear. According to the guidelines by the Japanese Circulation Society and the Japanese Association of Cardiac Rehabilitation, following items are relative contraindications for exercise training: exacerbation of HF symptoms (such as respiratory distress, fatigability, edema, pulmonary congestion) within the past 3 days, unstable angina, severe aortic stenosis, untreated exercise-induced severe arrhythmia, and/or acute systemic disease or fever, etc [10,12]. Therefore, if patients had one or more of these relative contraindications, that may have led to limitations in the amount of rehabilitation provided. As a parameter of severe arrhythmia, we assessed the proportion of implantable cardioverter-defibrillator (ICD) or cardiac resynchronization therapy defibrillator (CRT-D) use, but there was no significant difference among the 3 groups. We were not able to further investigate if any of the other relative contraindications were present in our patients and whether they affected the ADRT. On the other hand, older age, poor nutrition, and cognitive dysfunction may also be related to shorter ADRT and are also highly associated with frailty [30]. Rehabilitation staff should play a key role in identifying these patients to provide preventative multifactorial interventions and recovery care to preserve or restore their functional independence [31,32].

### Limitations

The present study has several limitations. First, the optimal contents or amount of rehabilitation during hospitalization is unclear. Further prospective studies are needed to determine optimal contents and amount of rehabilitation to prevent IADL decline during hospitalization. Second, relationship between ADRT and IADL decline was the focus of this study, and the effect of the timing (ex. early or late in the hospital stay) is unknown. Third, pre-hospitalizatoin NCGG-ADL was evaluated after admission, and thus the presence of recollection bias cannot be denied. However, this risk was minimized through excluding patients with severe cognitive dysfunction. Forth, because most activities listed in the NCGG-ADL scale are related to home tasks, there is a possibility that it could not accurately measure the IADL level of hospitalized patients. However, the NCGG-ADL is reported to evaluate not only the actual activity level achieved in daily living, but also the level of potential activity (capability ADL/IADL) [13,33,34]. Additionally, the physical therapists in our study carefully assessed each patient’s physical function, ADL, and cognitive function at discharge, which was taken into account when determining the NCGG-ADL score. Fifth, though we performed a multivariate analysis adjusting for investigable confounding factors, we cannot exclude the possibility that IADL decline was affected by other factors, such as psychological aspects. As depression and/or delirium are one of the well known factors of developing HAD, further studies might need to include psychological aspects as necessary. Sixth, patients in the short group may already have impaired physical function and ADL at the time of admission, and there was a possibility of poor recovery potential. Seventh, ADL and SPPB scores are data at the time of discharge, it is unclear whether the degree of recovery in ADL and physical function differed due to the short frequency and duration of rehabilitation provision as determined by physical therapists and physicians. Eighth, it is unclear whether the difference in rehabilitation time was due to patient factors or factors on the rehabilitation provider side in this study. Finally, this study was observational study so that it is difficult to represent the superiority of long groups. Intervention studies are needed to show the benefit of high frequency and volume of rehabilitation in the future.

## Conclusions

The present study demonstrated that 21% of older HF patients had IADL decline during hospitalization and short of ADRT may be associated with the risk of IADL decline during hospitalization in older patients with HF.
